# Recent technical advancements and clinical applications of MR-guided radiotherapy in lung cancer treatment

**DOI:** 10.3389/fonc.2025.1622060

**Published:** 2025-07-01

**Authors:** Chi Ma, Xiao Wang, Ke Nie, Zhenyu Xiong, Keying Xu, Ning Yue, Yin Zhang

**Affiliations:** Department of Radiation Oncology, Rutgers Cancer Institute, New Brunswick, NJ, United States

**Keywords:** magnetic resonance-guided radiotherapy (MRgRT), adaptive therapy, lung cancer, artificial intelligence, radiomics, lung SBRT

## Abstract

Magnetic resonance-guided radiotherapy (MRgRT) represents a significant advancement in lung cancer treatment, integrating non-ionizing high-resolution magnetic resonance imaging (MRI) with linear accelerators to enable real-time tumor visualization and adaptive treatment planning. This review highlights recent advancements in MRgRT technology and explores its clinical applications, particularly in managing lung cancer patients. MRgRT has proven particularly advantageous in stereotactic body radiotherapy (SBRT) treatment for lung cancer, where motion management is critical due to respiratory-induced tumor motion. Real-time tumor monitoring and online adaptive plan modifications ensure target accuracy, reduce margins, and mitigate radiation-induced toxicity. Additionally, MRgRT could potentially allow multileaf collimator (MLC) tracking to further improve the treatment efficiency. Recent technological innovations, including AI-powered auto-contouring algorithms, deep-learning (DL) based prediction models, and adaptive treatment strategies, further optimize MRgRT by improving workflow efficiency and reducing treatment time. Despite these benefits, the widespread adoption of MRgRT is challenged by high infrastructure costs, prolonged treatment time, and the need for specialized expertise. Ongoing research is addressing these challenges through workflow optimization, remote treatment models, and AI-driven decision support systems. As MRgRT technology continues to evolve, its integration with functional imaging, radiomics, and adaptive protocols is expected to expand its applications beyond lung cancer treatment. MRgRT represents a paradigm shift in precision oncology, delivering personalized care. Future research and prospective clinical trials should be warranted to generate high-quality clinical evidence supporting MRgRT’s clinical adoption for lung cancer patient management. As these advancements progress, MRgRT is poised to transform the future of lung cancer treatment.

## Introduction

Lung cancer remains a leading cause of cancer-related mortality worldwide, accounting for over 1.8 million deaths annually (1). Non-small cell lung cancer (NSCLC) represents approximately 85% of all lung cancer cases (2). Despite advancements in systemic therapies, radiotherapy continues to play a critical role in lung cancer patient management. Advanced radiotherapy techniques, such as stereotactic body radiotherapy (SBRT) and image-guided radiotherapy (IGRT), have significantly improved local control and reduced toxicity (3–5). However, there are still challenges in lung cancer radiotherapy treatment, including intrafractional motion, anatomic changes, and dose delivery to critical organs (6–8).

Magnetic resonance-guided radiotherapy (MRgRT), such as the MRIdian Viewray system and the Elekta Unity system, has emerged as a promising solution to address these challenges. By integrating real-time magnetic resonance imaging (MRI) with a linear accelerator (linac), MRgRT enables continuous visualization of target volumes and critical structures during treatment without additional ionizing imaging dose (9, 10). The ability to perform real-time visualization and adaptive radiotherapy (ART) using MRgRT has proven beneficial in improving target dose coverage while protecting surrounding organs at risk (OARs) (11–17), especially in complex clinical scenarios such as ultracentral lung tumors (ULT) and tumors exhibiting significant or irregular motion during respiration (18–20). MRgRT allows for dose escalation strategies that were previously infeasible with conventional modalities (13, 21).The growing body of evidence from clinical trials and institutional experiences highlights the potential of MRgRT to redefine standards of care in lung cancer radiotherapy. Moreover, the incorporation of artificial intelligence (AI) (22, 23) and radiomics (24–27) into MRgRT workflows offers new opportunities to personalize and optimize treatment by assessing biological and anatomical changes throughout the treatment course.

This review explores the latest technological advancements, clinical applications, and emerging trends in MRgRT for lung cancer treatment. We discuss the advantages, limitations, and future directions of MRgRT, with a focus on its role in enhancing treatment precision, reducing toxicity, and improving clinical outcomes for lung cancer patients.

## Technological advancements in MR-guided radiotherapy in lung cancer treatment

### MR-linac system

The two most commonly used MR-linac platforms are the Elekta Unity (10, 28) and the ViewRay MRIdian (29, 30). ViewRay MRIdian uses a split 0.35T MRI scanner. The ViewRay system was equipped with a cobalt-60 source in earlier versions and started first clinical use at Washington University in 2014 (29). The system was later upgraded with linac technology in the new MRIdian version, which was introduced in 2017 (30, 31). Elekta Unity uses a 1.5T MRI scanner integrated with a 7 MV flattening filter-free (FFF) linac. This system started clinical use in 2018 (32), after the first clinical cases treated in 2017 at UMC Utrecht in close collaboration with Elekta and Philips with their prototype MRI-Linac (10).

Both platforms provide continuous cine-MRI during treatment, enabling clinicians to visualize and track tumor motion in real-time (33). This capability is particularly advantageous in lung cancer treatment, where respiratory-induced motion can significantly affect target positioning. With the instantaneous tumor motion information from MRI images, these systems could potentially achieve real-time multileaf collimator (MLC) adjustments following target motion (34), enhancing treatment accuracy and efficiency and reducing the need for large treatment margins. Additionally, the automatic beam gating became possible on both systems with the cine-MRI images and this function ensures that radiation is delivered only when the tumor is within the predefined target boundary, significantly reducing the risk of damage to surrounding tissues while maintaining adequate tumor dose. Both platforms offer advanced imaging sequences, including T2-weighted and diffusion-weighted imaging (DWI), enhancing soft-tissue contrast and functional imaging capabilities. These features allow clinicians to assess tumor response and identify functional changes.

### Online adaptive radiotherapy

Online ART is one of the most significant advancements and cornerstones of MRgRT (11, 35). Both ViewRay MRIdian and Elekta Unity systems have built-in TPS and treatment planning workflow that enables online ART (29, 32). Traditional radiotherapy relies on a treatment plan generated prior to the first session and applied across the entire course of treatment. When anatomical changes are observed, a revised treatment plan will be generated in an offline manner. In contrast, online ART allows clinicians to modify treatment plans daily based on changes in tumor size, position, and the surrounding anatomy. This is achieved through online MR imaging, contouring based on the anatomy of the day, and re-optimization with patient on the treatment table within the same session. This capability is particularly beneficial for lung cancer patients, whose tumors can shift substantially due to respiratory motion, weight loss, or disease response or progression (13).

Clinical implementation of online ART has demonstrated the ability to reduce planning target volume (PTV) margins and spare organs at risk (OARs) more effectively (36). Modern MRgRT platforms now can potentially incorporate AI-assisted auto-contouring, auto-registration, and dose prediction tools that significantly accelerate the adaptive process (22, 23). A growing number of studies have validated the feasibility, safety, and clinical benefits of daily adaptation, including improved tumor control and reduced toxicity (17, 37, 38). Workflow enhancements and the increasing availability of trained personnel are making online ART more accessible for routine use. At the same time, integration with radiomics and biological imaging may enable biologically adaptive planning, further personalizing treatment and improving outcomes.

With existing MRgRT platforms, clinical focus on lung cancer treatment has primarily focused on stereotactic treatment (39), which has been proven to be effective in local control of early-stage lung tumors. SBRT is the recommended standard treatment option for medically inoperable early-stage non-small cell lung cancer (40) and has shown effectiveness in treating oligometastic disease in lung (41). Existing evidence supports the treatment of peripherally located tumors, where the tumor is away from critical OARs such as major airways.

**Figure 1:**
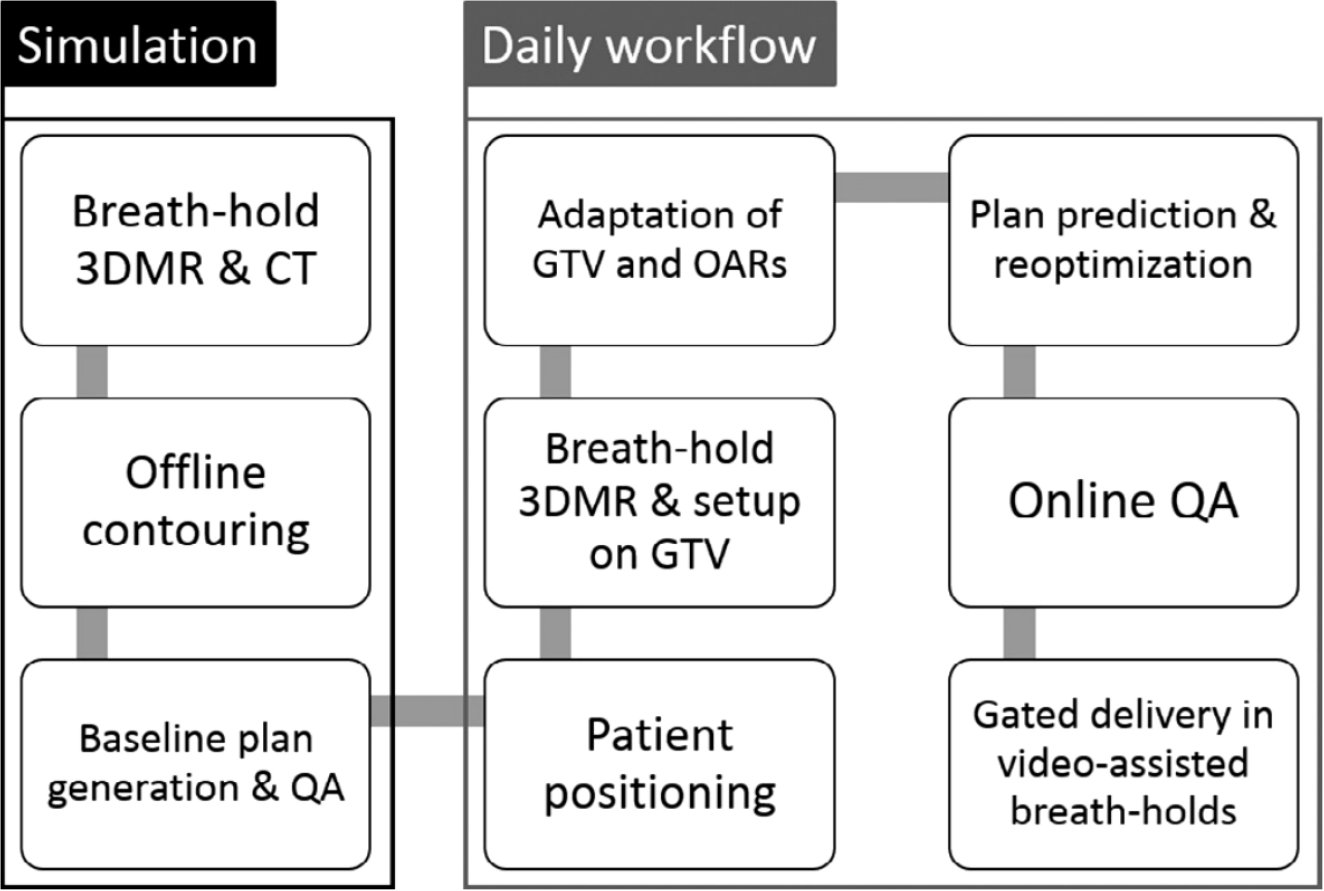
Schematic visualization of the SMART procedure for lung tumors. During simulation, patients undergo a breath-hold 3DMR and CT scan. A baseline treatment plan, to be used for daily plan adaptation, is generated offline. The daily workflow then consists of MR-guided patient setup, online plan adaptation including on-table QA, and gated breath-hold delivery. Reprinted with permission from ref. 14. Copyright 2020, Elsevier.

The MRgRT platform, with superior soft-tissue contrast and the ability to provide online adaptive workflow, yields excellent clinical results when compared to traditional SBRT treatment without adaptive workflow (42). For example, Finazzi et al. proposed the stereotactic MR-guided adaptive radiation therapy (SMART) approach (43) and their study including 25 patients with peripheral lung lesions demonstrated great clinical results using the SMART approach (14). An example schematic visualization of the SMART workflow is shown in **Figure 1** (14). In this gated breath-hold study, the PTVs generated on daily breath-hold 3D MRI were on average only 53.7% of the volume of the ITV-based PTV generated using 4DCT. In addition, the online ART process improved prescription dose coverage of the PTV from a median of 92.1% in predicted plans, to 95.0% in reoptimized ones. A single-institution retrospective study investigated the clinical outcomes of SMART in primary tumors and lung oligometastases (17). This study included 64 patients with 92 lung tumors with 80.4% of the tumors peripherally located. The follow-up study shows 1-year and 3-year local progression-free survival rates of 96.3 and 86.4% respectively without ≥grade 3 toxicity.

SBRT treatment of central and ultracentral tumor (ULT) has been controversial, due to the toxicity caused by the proximity of target to major airways (44). The centrality of tumor was defined according to RTOG 0813 criteria, which include tumors located within 2 cm of the proximal bronchial tree or those with a planning target volume (PTV) in contact with the mediastinal or pericardial pleura. Additionally, tumors were sub-classified as “ultracentral” if the gross tumor volume (GTV) directly abutted the proximal bronchial tree or trachea (45).

MRgRT has also garnered interest in addressing the challenges associated with SBRT for central and ultra-central lung tumors. The *in silico* studies conducted by Henke et al. on the treatment of central tumors within a simulated MRgRT online ART environment have demonstrated promising results, showing improved sparing of OARs compared to non-adaptive treatment approaches (46).

**Figure 2:**
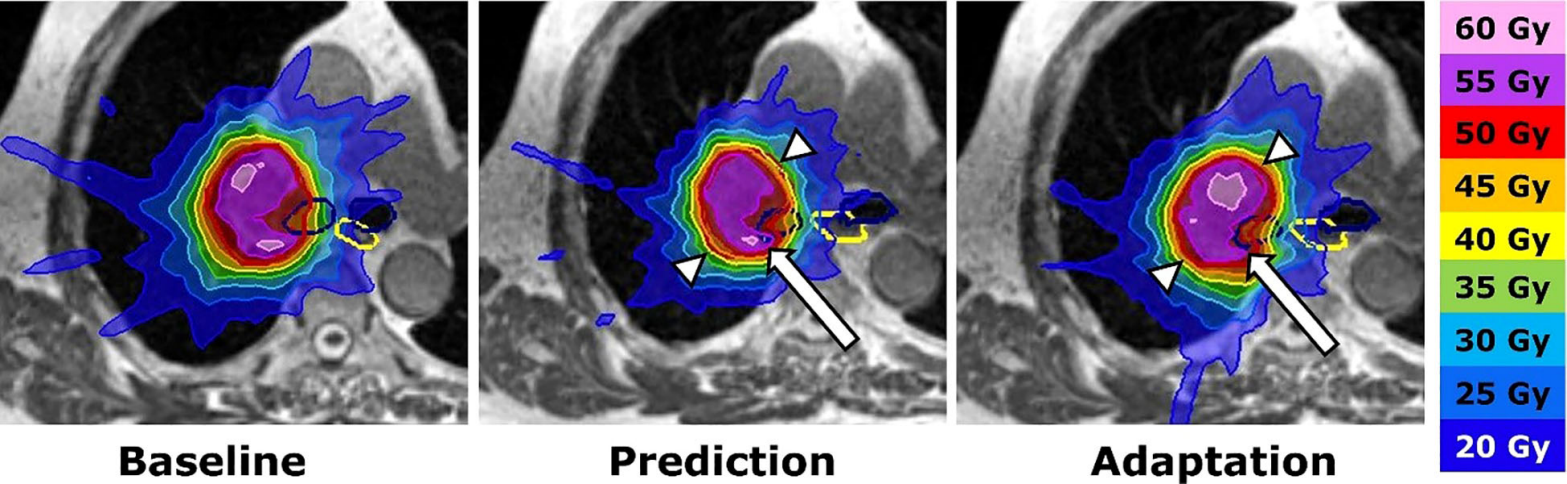
Adaptation procedure. SMART of an ultracentral lymph node metastasis. Left: Baseline plan with contours of the planning target volume (PTV: red), main stem bronchi (dark blue) and esophagus (yellow). Middle: The dose distribution of the baseline plan was predicted on the daily anatomy (fraction 7). The dose declines at the anteromedial and posterolateral PTV border (white arrowheads), while the right main bronchus receives an overdose (white arrow). Right: Plan adaptation optimizes the PTV coverage and avoids overdoses inside the right main bronchus. Reprinted with permission from ref. 44. Copyright 2023, Elsevier.

Since then, various studies have been proposed to investigate the characteristics of SMART approach treating ULT. A prospective study by Regnery et al. investigates the long-term outcomes and safety of SMART in treating ULT tumors (44). In this study, 16 ULT underwent SMART workflow, online ART was performed in 91% of fractions. After a median follow-up of 23.6 months, the overall survival rate was 67%, and the 2-year local progression rate was 7%. Their study supports SMART as potentially effective treatment of ULT. An adaptation example where original planning objectives were violated is demonstrated in **Figure 2**. The same group published another study posting results of the MAGELLAN trial (20), which aims to find the maximum tolerated dose of MR-guided SBRT of ULT. The analysis focused on the proximal bronchial tree (PBT) dose on 19 patients with ULT treated with SMART (18). They found that both intrafractional breathing motion and interfractional translations may impact doses to the PBT during SBRT of ULT. SMART protects the PBT from overdoses and maintains high PTV coverage, while non-SMART approach appears safe with advanced breathing motion management and planning organ at risk volume (PRV) but yields inferior PTV coverage.

In addition to SBRT approach, hypofractionated approach has been investigated by La Rosa et al. in ULT (19, 47). In this institutional study, thirteen patients with 14 ULT tumors were treated with 60 Gy in 15 fractions using MRgRT, with daily online adaptation for all 195 delivered fractions. The study achieved a 92.3% crude in-field locoregional control with no reported grade 3 or higher treatment-related toxicities (CTCAE v5.0), showing promise of online ART with MRgRT in the context of non-SBRT setting.

At the moment of this writing, most of the studies described in this review have been done with 0.35 T ViewRay systems (MRIdian MR-Linac or older Co-60 system).

**Figure 3:**
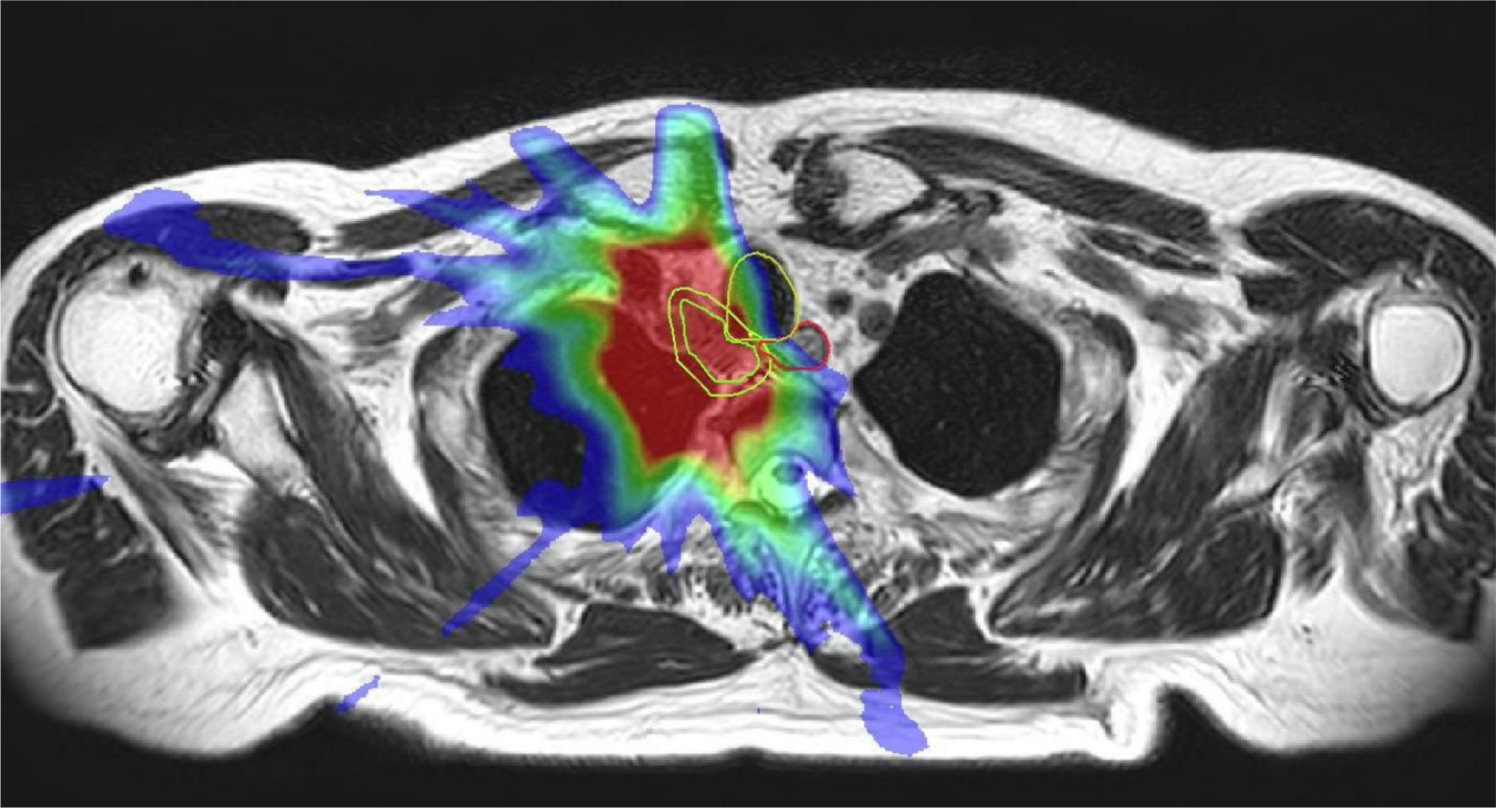
Online MRI and physical dose distribution of a patient with a mediastinal lymph node metastasis of a SCLC in whom a 58% decrease in ITV occurred. The dose distribution of the original plan is projected on the reduced target size at the 12th fraction The ITV (inner green line) and OARs (oesophagus, trachea, spinal cord) within a 3 cm expansion (yellow) around the ITV were modified each fraction by a radiation oncologist. Reprinted with permission from ref. 37. Copyright 2024, Elsevier.

Due to relative late introduction to the clinic, reports with large number of patients on the 1.5 T Elekta system have been scarce. Merkel et al. reports the first clinical experiences of SBRT for ULT using Elekta Unity. The clinical data from 10 patients were collected from 2020-2022 (37). In this study, online ART was performed before each fraction using a T2-weighted 3D MRI acquired during free breathing. Adaptation improved ITV coverage in 34% of the cases, while 14% experienced reductions due to OAR dose constraints. **Figure 3** demonstrated a case where decrease in ITV volume occurred in this study.

### Motion management systems

Tumor motion is a critical challenge in thoracic radiation treatment, particularly for lung cancers that are subject to continuous movement due to respiration and cardiac activity. In addition, the dose conformity of IMRT and VMAT increases the need for respiratory motion management (48).

MRgRT provides powerful tools for motion management through real-time imaging and tracking (49). Motion tracking system from ViewRay MRIdian has been enabling breath-hold gating treatment technique, by taking 2D cine MR images at a rate of 4 fps (33, 50). The system was recently upgraded with compressed sensing and additional motion tracking algorithms, which enabled respiratory gating at 8 fps, with better in-plane resolution and larger matrix size (51). However, planar images with in-plane analysis can only evaluate one-dimensional motion, for example, motion along the SI direction (51). For more complex three-dimensional geometries with motion in various directions, a single imaging slice at high frame rate may not be sufficient.

Recently, Elekta introduced the comprehensive motion management (CMM) system to Unity MR-Linac (52). With CMM, 3D anatomical motion is monitored in two planes (sagittal and coronal) during treatment using a live 2D MR cine balanced turbo field echo (bTFE) imaging sequence acquired with 170 ms temporal resolution per plane, or a temporal resolution of 340 ms two planes combined. 3D-2D registration enables contour tracking in three directions. Multiple automatic gating options are provided in this CMM system. Respiratory gating techniques within CMM include expiration gating, average gating, and breath hold. A non-respiratory gating technique is also provided that can automatically pause the beam if the target moves outside of a user defined threshold in the bTFE cine MRI images (53). This motion management system also allows for correction of tumor drift and bulk shifts. A recent commission study demonstrated CMM system has accurate target positioning prediction with a low beam delivery latency resulting in negligible impact on the overall gated dose distribution (54). The bi-plane approach of motion tracking also has limitations. One noticeable limitation is that the current CMM can only monitor/measure motion for one target belonging to both coronal and sagittal planes of cine MRI (54).

### MLC tracking

MLC tracking is a technique used to compensate for intrafraction motion during radiotherapy taking an input from a real-time target position monitoring system and adjusting the radiation beam using the MLC to improve the alignment between the beam and the target (55, 56). This technique has been actively explored in various radiation therapy systems including MRgRT (55).

One research group focused on MLC tracking with MRI-linac with IMRT and VMAT plans on lung plans (34, 48). This group later refined their methods by using a moving dosimetry phantom platform with new leaf sequencer and dose optimization algorithm (57). Their most recent results showed VMAT plans maintained clinical dose constraints and achieved an average local gamma pass rate of 93% under motion with MLC tracking, significantly outperforming untracked deliveries. The system demonstrated a high delivery efficiency of 83%, a notable improvement over gated IMRT. However, current implementation of real-time tumor tracking in MRgRT is associated with approximately a 300–350 ms system latency, which is still higher than the desired latency of 150 ms to safely eliminate ITV in VMAT delivery (58).

### Delta radiomics

The variation of radiomic features across different imaging time points during treatment—referred to as delta radiomics—has been proposed in the literature as a predictive tool in various oncologic settings, showing potential for forecasting both treatment response and toxicity outcomes (59, 60).

Proof-of-concept delta radiomics studies have been conducted with 0.35T MRgRT scans on ViewRay MRIdian system with different disease sites such as pancreatic cancer (24) and rectal cancer (25, 26).

A 2024 study focused on lung cancer evaluated 47 SBRT treatments in 39 lung cancer patients using ViewRay MRIdian MR-Linac and analyzed MR delta radiomic features extracted via Pyradiomics (27). These features spanned seven classes, including shape, histogram, and texture descriptors. The researchers focused on stable and non-collinear features, narrowing down from 107 to 15 core metrics. The study demonstrated the feasibility of delta radiomics with MRgRT for lung cancer and underscored its potential in adaptive radiotherapy, where treatment is tailored in real time based on biological response.

### AI integration

AI has been the core of technical innovations in recent years, and the influence has been quickly adapted in the healthcare sector, especially in the technology-driven field of radiation oncology. AI-driven solutions have been increasingly incorporated into auto-segmentation (61) and advanced imaging techniques within adaptive planning framework, these advances aim to address MRgRT’s main limitations such as extended session durations and move closer to treatment timeframes typical of conventional radiotherapy.

Precise delineation of target volumes and OARs is essential for effective treatment planning and adaptation, yet the process is time-consuming and prone to inter-observer variability. Although commercial tools for automatic contouring exist for CT-based workflows, dedicated solutions tailored to MRI are still required to fully support online MRgRT. Automatic DL-based contouring has been explored for MR images from MRgRT systems in abdomen and pelvis regions, no studies are currently available that specifically focus on the thoracic region (62–65).

4D imaging is another key field of needs and advances driven by AI in MRgRT, namely time-resolving 3D MRI (4D-MRI) and synthetic 4D-CT. The need for 4D-MRI primarily stems from the requirement for MRI images with high temporal and spatial resolution for online adaptive therapy (66, 67). 4D-MRI has been incorporated into abdomen SBRT workflow (67) and liver SBRT in a prospective non-randomized patient study (66, 68) on Elekta Unity with 1.5T MRI scanner. In both studies, two pre-beam 4D-MRIs are acquired, one for the purpose of adaptive re-planning, then another one immediately before beam-on for additional verification. In the liver SBRT study, one additional 4D-MRI is acquired intra-fraction to provide information regarding if additional adaptation is required (66). This approach, independent from vendor provided motion management, has its limitation especially for intra-fraction monitoring. For each 4D-MRI, 4min acquisition time and 40s reconstruction time are needed. This does not meet the sub 500ms latency recommended to capture breathing-induced motion, which is key in lung cancer treatment. Although deep learning based methods to increase spatial resolution on sparsely sampled high temporal resolution are attempted by various research studies (69, 70), To date, MRgRT workflow incorporating 4DMRI has not been established with lung cancer patients.

The need for synthetic CT images in MRgRT stem from the desire for accurate dose calculation in the absence of CT in pure MRI environment with the paradigm of MRgRT, and ultimately, an MRI-only treatment planning framework. For lung cancer treatment on MRgRT, more accurate intrafractional dose accumulation required synthetic 4D-CT datasets, which can be generated from 4D-MRI images (72). Orthogonal cine MRI generated during beam delivery at clinical MR-linacs can also be utilized to generate synthetic 4D-CT datasets. Using a propagation method (71), continuous time-resolved estimated synthetic 4D-CTs were generated for dose reconstruction of lung tumor treatments using ViewRay MRIdian MR-linac (72).Various attempts have been conducted at using AI to generate synthetic CT. These DL networks-based methods cover clinical sites such as brain, H&N and abdomen and pelvis regions. However, no studies solely focused on synthetic 4D-CT with AI approaches (22).

## Challenges and future directions

While MRgRT presents numerous promising advancements in lung cancer treatment, several challenges remain to be addressed to fully harness its potential and achieve widespread clinical adoption.

### Technical and logistical challenges

One of the significant barriers to broader MRgRT implementation is the complexity and resource-intensive nature of the technology.MR-Linac systems require substantial investment in infrastructure, specialized equipment, and highly trained personnel. The integration of MRI with linacs necessitates specialized facilities designed to minimize magnetic interference and accommodate safety protocols associated with strong magnetic fields.

MRI of the lung presents unique challenges due to the inherently low proton density of the pulmonary parenchyma and the presence of numerous air–tissue interfaces, which cause rapid signal decay and susceptibility artifacts. As a result, conventional MRI sequences often yield poor SNR in lung tissue, making it difficult to visualize fine parenchymal structures. In diagnostic radiology, this limitation is addressed through the use of specialized pulse sequences such as ultra-short echo time (UTE) (73, 74), which are designed to capture signal from tissues with very short T2* relaxation times. However, in the context of MRgRT, the clinical imaging requirements differ. The goal of lung MRgRT is not primarily focused on detailed parenchymal imaging, but rather accurate localization of gross tumor volumes (GTVs), surrounding soft tissues, and organ motion tracking. MR-linac systems optimize for tumor and boundary visualization using sequences like TRUFI (56) or other balanced SSFP sequences (52), which provide sufficient contrast for target delineation and motion management even without parenchymal detail. Thus, while the limited lung parenchymal signal remains a technical constraint, it does not critically impair the clinical efficacy of MRgRT. Nevertheless, ongoing research into MR pulse sequence development, including UTE applications on MR-linacs, may further enhance imaging performance in thoracic radiotherapy.

Note that other online ART platform, such as Ethos system (Varian Medical Systems, Palo Alto, CA, USA) that uses daily cone beam computed tomography (CBCT) scans in the online ART workflow, shows potential to treat lung tumors in recent feasibility study (48, 49). The visualization of small lung tumors with large motion, especially those located close to diaphragm, could still be challenging for both CBCT-based ART and MR-based ART modalities, due to different reasons. Compared with MR-base ART, the CBCT-based ART currently does not allow real-time visualization of lung tumors during beam delivery and adds extra ionization imaging dose (49).

Recent studies have highlighted that the introduction of real-time tumor tracking and online ART significantly increases the procedural complexity and may initially decrease departmental throughput, creating practical challenges for clinical implementation. Furthermore, MR-Linac systems often require extensive quality assurance (QA) protocols to maintain the accuracy and consistency of MR component in addition to regular linac QA, adding further logistical complexity (75).

Addressing these technical barriers will require innovations such as improved system integration, streamlined imaging techniques, faster and more efficient adaptive software, and optimized patient workflow strategies. Collaboration between equipment manufacturers, clinical experts, and healthcare providers will be crucial to overcoming these challenges and fostering broader adoption of MRgRT.

## Treatment time and patient compliance

Extended treatment time, inherent to online ART sessions, poses challenges related to patient comfort, compliance, and clinical throughput (76). Studies utilizing MR-Linac systems have demonstrated treatment durations significantly longer than conventional radiotherapy, with single fraction sometimes exceeding an hour due to the necessity of repeated imaging, adaptive replanning, and gated treatment processes (77). Lengthy treatment sessions can result in increased patient discomfort, potential motion during treatment, and reduced patient compliance. Future efforts should focus on reducing treatment durations through advancements in rapid imaging techniques, accelerated adaptive planning algorithms, and improved patient immobilization and comfort strategies. Enhanced patient education and engagement protocols could further improve patient experience and compliance during treatment.

## Standardization and quality assurance

The variability in MR imaging protocols, adaptive treatment planning strategies, and workflow practices across different institutions highlights the need for standardization and QA guidelines. Initiatives led by professional societies are underway to develop detailed guidelines for standardized imaging and adaptive planning protocols (78, 79). Rigorous training programs for radiation oncologists, medical physicists, and radiation therapists in standardized MR-guided techniques can further enhance consistency in clinical outcomes.

### Clinical evidence and prospective trials

Although retrospective studies and early clinical experiences demonstrate MRgRT’s potential benefits, robust prospective clinical trials and long-term outcome data are needed to conclusively establish its clinical efficacy, safety profile, and comparative effectiveness versus conventional radiotherapy approaches (20).

In a recent narrative review, Cheng et al. summarized ongoing MRgRT lung clinical trials, emphasizing the critical need for prospectively collected data to evaluate outcomes such as overall survival, local control, and toxicity profiles (80). These trials are particularly important in high-risk clinical scenarios—such as ultra-central tumors (ULT) or re-irradiation cases—where prospective evidence is needed to define safe and effective dose regimens and to characterize associated toxicity (18).

Future research should focus on conducting multi-center, randomized controlled trials to generate high-quality evidence supporting MRgRT’s clinical adoption and incorporation into standard clinical guidelines. Establishing multicenter collaborative networks and patient registries dedicated to MRgRT research will facilitate large-scale data collection, rigorous outcome analysis, and meaningful comparisons across clinical settings (16). These coordinated efforts will ultimately help facilitate the integration of MRgRT into standardized management of lung cancer patients.

## Conclusion

MRgRT has emerged as a transformative technology in lung cancer treatment, offering daily online adaptive capabilities and unprecedented non-ionizing real-time imaging during radiation treatment. Its application in treating lung cancer patients has demonstrated superior dosimetric and clinical outcomes. As technological advancements continue, including advanced motion management techniques, AI-driven adaptive planning and workflow optimization, and delta radiomics, MRgRT is poised to be further integrated into standard oncological practice.
